# Research Progress of Food-Grade High Internal Phase Pickering Emulsions and Their Application in 3D Printing

**DOI:** 10.3390/nano12172949

**Published:** 2022-08-26

**Authors:** Chao Wu, Zhe Liu, Lanyi Zhi, Bo Jiao, Yanjie Tian, Hongzhi Liu, Hui Hu, Xiaojie Ma, Marc Pignitter, Qiang Wang, Aimin Shi

**Affiliations:** 1 College of Food Science and Engineering, Hebei Agricultural University, Baoding 071001, China; 2 Institute of Food Science and Technology, Chinese Academy of Agricultural Sciences/Key Laboratory of Agro-Products Processing, Ministry of Agriculture, Beijing 100193, China; 3 Department of Physiological Chemistry, Faculty of Chemistry, University of Vienna, 1090 Vienna, Austria

**Keywords:** HIPPE, solid particle, interfacial film, rheology, 3D printing

## Abstract

High internal phase Pickering emulsion (HIPPE) is a type of emulsion stabilized by solid particles irreversibly adsorbed on an interfacial film, and the volume fraction of the dispersed phase (*Φ*) is larger than the maximum packing volume fraction (*Φ*_max_). Proteins, polysaccharides, and their composite particles can be used as good particle stabilizers. The contact angle can most intuitively demonstrate the hydrophilicity and hydrophobicity of the particles and also determines the type of emulsions (O/W or W/O type). Particles’ three-phase contact angles can be adjusted to about 90° by compounding or modification, which is more conducive to emulsion stability. As a shear thinning pseudoplastic fluid, HIPPE can be extruded smoothly through 3D printer nozzles, and its high storage modulus can support the structure of printed products. There is huge potential for future applications in 3D printing of food. This work reviewed the biomacromolecules that can be used to stabilize food-grade HIPPE, the stabilization mechanism of the emulsions, and the research progress of food 3D printing to provide a reference for the development of advanced food products based on HIPPE.

## 1. Introduction

Pickering emulsion is a type of stable emulsion formed by the hydrophilic and hydrophobic solid particles that are irreversibly adsorbed onto the oil–water interfacial film [1]. Compared with tradition emulsions using surfactants, it has obvious advantages: reduced harmful effects of emulsifiers on humans, reduced production costs, environmental friendliness, etc. Pickering emulsions can encapsulate active ingredients such as curcumin, lycopene, and other fat-soluble nutrients and serve to improve digestive absorption and bioavailability [2].

High internal phase Pickering emulsion (HIPPE) is an emulsion in which the volume fraction of the dispersed phase (*Φ*) is larger than the maximum packing volume fraction (*Φ*_max_) [3,4]. HIPPE has the advantages of high stability, good safety, and excellent resistance to Ostwald ripening. It is widely used in wastewater treatment, cosmetics, pharmaceuticals, and the food sector [5]. For example, in the oil field, the high internal phase emulsion cannot only ensure a high carrying capacity but can also reduce the risk of leakage during transport by utilizing its unique rheological properties. It has good application value in the oil field and also provides ideas to simplify the separation process of oil and water later on. HIPPE has been found in research to have higher loading and biological accessibility than conventional emulsions in delivering lipid-soluble active substances [6] and has a broad future in the development of drugs and fortified foods containing nutrients. In addition, due to its safety, thermal stability, freeze–thaw stability, and rheological properties, HIPPE can be used as a substitute for some hydrogenated vegetable oil and also has a great potential in the development of advanced food products, such as ice cream, salad dressing, and mayonnaise [7,8], which is a future research hotspot and challenge in the field of food colloids.

Biomacromolecules have many advantages. They are extensively sourced, renewable, and biodegradable and, hence, often used to stabilize HIPPE. Macromolecules of biological origins are mainly divided into proteins (vegetable proteins, animal proteins, etc.), polysaccharides (starch, cellulose, etc.), and their composite particles. However, due to the poor wettability of most solid particles, it is often necessary to modify the particles to improve their wettability and amphipathy so that they can better adsorb at the oil–water interface and form steric hindrance, thus stabilizing HIPPE. In addition, food-grade HIPPE also has excellent rheological properties; research has proven that the energy storage modulus of HIPPE is always greater than the loss modulus in frequency scan tests, indicating a good elastic gel strength. Meanwhile, the apparent viscosity tends to decrease as shear rate increases, indicating that HIPPE is a pseudoplastic fluid with shear thinning behavior [9]. As a new technology, 3D printing is widely used in the aerospace and medical fields, such as for realizing the customization of fine medical models, printing high-temperature parts with metallic powder, and so on [10,11,12]. Additionally, 3D printing has boomed in the food sector in recent years. Because of the above characteristics, there has been a preliminary study on applying HIPPE to food 3D printing, which has expanded the application range of the emulsion system in emerging foods.

This work reviewed the biomacromolecules that can be used to stabilize food-grade HIPPE, such as vegetable proteins, animal proteins, protein microgel particles, and polysaccharides and their composites, and the stabilization mechanism of the emulsions and their applications in food 3D printing. We aimed at looking into the future development direction and providing a reference for 3D printing product development based on food-grade HIPPE.

## 2. HIPPE Stabilized by Solid Particle

The widely sourced vegetable and animal proteins as well as polysaccharides can be used as solid particle stabilizers for HIPPE.

### 2.1. Protein Particles

As natural biomacromolecules, proteins are important nutritional components in food. They have a good emulsification property, interfacial activity, and biodegradability and are often used as emulsifiers to stabilize food-grade HIPPE [13]. We enumerated the high internal phase Pickering emulsion stabilized by protein particles, as shown in Table 1.

Vegetable proteins are widely distributed and inexpensive. Compared with animal proteins, they have the advantages of accessibility and sustainability, which can meet the needs for vegetarians and those who like plant food. Vegetable proteins have good emulsification and gelation properties and have been proven to be excellent stabilizers for high internal phase emulsions [14]. However, natural proteins also have some limitations in stabilizing high internal phase emulsions since droplet flocculation and agglomeration may occur [15]. Factors such as high-pressure treatment, heat treatment, ultrasonic treatment, pH, and ions can have an effect on the emulsification properties of proteins. High-pressure treatment can rearrange the protein structure, which reduces the particle size of the proteins, reduces flocculation, and reduces the particle diameters; thermally induced proteins have higher surface hydrophobicity and lower surface charge [16,17]. Ultrasound affects the secondary and tertiary structure of proteins, resulting in an increase in protein solubility, a decrease in particle diameters, and also an increase in the contact angle, thus improving the emulsification capacity [18,19]. Acid-base properties can regulate the protein morphology, which further affects the microstructure, physical stability, and rheological properties of high internal phase emulsions [20]. Through ion induction, proteins can produce a salt bridge effect, become hydrophobic, and act as hydrogen bonds. In the future, protein denaturation can be induced by different physicochemical methods to make protein particles adsorbed at the oil–water interface, thus improving the stability of high internal phase emulsions and expanding the application of vegetable proteins in the food industry.

Lactoprotein, ovalbumin, gelatin, meat proteins, and other animal proteins [21] have excellent gelling, foaming, and emulsification properties and are important in the preparation of food-grade high internal phase emulsions. However, because of the poor stability and solubility, this will be the key factor to be overcome in future research. The pH value and homogeneous parameters of protein particles have a significant influence on droplet size during the preparation of high internal phase emulsions; it has been found that the droplet size decreases linearly with the increase in pH and homogenization time of the particle solution, while it decreases exponentially with the increasing homogenization speed [22]. Moreover, changing the properties of milk proteins by heat treatment [23], ion treatment, and ultrasonic treatment can serve to better stabilize the high internal phase emulsions, which have a great potential of application in the food industry. However, the current research on the use of meat proteins as stabilizers for high internal phase emulsions is not sufficient, and future research can be conducted on the interfacial structure and stabilization mechanism of meat proteins for stabilizing high internal phase emulsions to fully explore the application potential of meat proteins in the emulsion system.

**Table 1:** Food-grade high internal phase emulsions stabilized by protein particles.

Protein Classification	Protein Type	Particle Preparation Method	Emulsion Preparation Method	*Φ*	Ref.
Vegetable protein	Prolamine	Dissolve protein in ethanol–water binary solvent to make protein solution, add acetic acid dropwise, and then shear at 6000 rpm for 10 min, and the ethanol is removed by rotary evaporation at 40 °C.	Mix with corn oil and shear at 20,000 rpm for 2 min to prepare the emulsion.	80%	[24]
	Soy lipophilic protein	Dissolve soy lipophilic protein in phosphate buffer, stir it for 24 h, then shear for 1 min after acid treatment.	Mix with soybean oil and shear at 10,000 rpm for 2 min to prepare the emulsion.	80%	[25]
	Soy lipophilic protein	Dissolve soy lipophilic protein in phosphate buffer and stir it for 24 h. Then, after acid treatment, prepare particles with ultrasound treatment of 350 W at 20 kHz.	Mix with soybean oil and shear at 10,000 rpm for 1 min to prepare the emulsion.	80%	[26]
	Quinoa protein isolate	Adjust pH value of quinoa protein isolate to 7.5, stir for 2 h, and then prepare particles with ultrasound treatment of 350 W at 20 kHz in the ice bath.	Mix with peanut oil and shear at 15,000 rpm for 2 min to prepare the emulsion.	80%	[20]
	Quinoa protein isolate	Stir quinoa protein isolate suspension for 24 h, mix vigorously after the pH value is adjusted to 7.0, and then prepare the particles through 500 bar high-pressure homogenization treatment (10 times).	Mix with soybean oil, add sodium azide as preservative, and then shear at 20,000 rpm for 5 min to prepare the emulsion.	80%	[16]
Animal proteins	Casein	Mix the casein solution, keep it overnight for thorough hydration, adjust pH value to 11 with KOH, stir for 1 h, and then adjust the pH value to 3.0 with HCl.	Mix with corn oil and add sodium azide and then shear at 80,000 rpm for 3 min to prepare the emulsion.	80%	[27]
	Gelatin	Add acetone dropwise into gelatin solution stir for 10 min, add glutaraldehyde solution for cross-linking, stir for 3 h under 50 °C, and centrifuge with 10,000× *g* for 20 min (3 times).	Mix with sunflower seed oil and shear at 13,500 rpm for 30 s to prepare the emulsion.	80%	[28]
	β-lactoglobulin	Keep the protein solution overnight under 4 °C, heat under 60 °C for 30 min, adjust pH value to 9.0, add genipin–ethanol solution to cross-link for 3 h, and then centrifuge with 20,000× *g* for 20 min (3 times).	Mix with soybean oil, add sodium azide, and then shear at 20,000 rpm for 2 min to prepare the emulsion.	75%	[29]
Protein microgel	Soy protein microgel	Fully hydrate the protein dispersion by keeping it under 4 °C overnight, prepare gel by heating it under pH 7.0 in the 80 °C bath, and then prepare particles by shearing at 10,000 rpm for 3 min.	Mix with sunflower seed oil and shear at 6000 rpm for 3 min to prepare the emulsion.	80%	[30]
	Soy protein microgel	Stir the protein for 30 min under 25 °C, heat the protein in bath to 90 °C, stir until the protein denatures, add glucono-δ-lactone (GDL) into the solution, form gel in 50 °C bath after 20 min, and then prepare particles through high-speed shear.	Mix with oil and shear at 12,000 rpm for 2 min to prepare the emulsion.	80%	[9]
	Peanut protein isolate microgel	Add transglutaminase (TG) enzyme in the protein solution to form gel and then prepare particles by shearing for 2 min at 10,000 rpm and high pressure of 100 MPa for 2 min.	Mix with oil and shear at 10,000 rpm for 2 min to prepare the emulsion.	87%	[7]
	Casein microgel	Adjust the solution pH value to 8, mix with glutaraldehyde, cross-link under 50 °C for 24 h, and dialyze for 2 d in deionized water.	Mix with olive oil and prepare the emulsion with hand-shaking method.	80%	[31]
	Gelatin microgel	The protein is swelled in 25 °C water for 60 min; then, stir it for 30 min under 60 °C.	Mix with soybean oil and shear at 10,000 rpm for 2 min to prepare the emulsion.	75%	[32]

**Figure 1:**
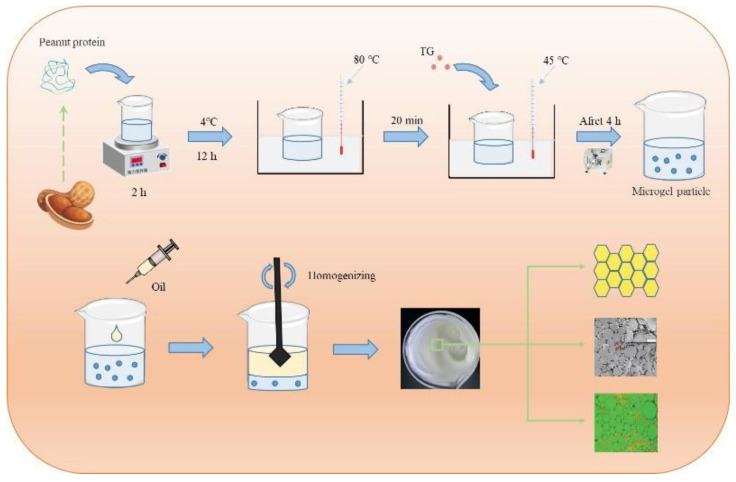
Schematic diagram of preparation of HIPPE by peanut protein microgel particles. Reprinted with permission from Ref. [7]. Copyright 2018 John Wiley and Sons.

Protein microgel particles are the products of protein modification, which is a three-dimensional network structure formed by the aggregation of protein molecules under the forces of hydrophobic interactions, van der Waals forces, hydrogen bonds, and electrostatic interactions [30]. The particles have unique physicochemical properties and structural characteristics. It has been reported that the acid-induced soy protein isolate microgel particles, heat-induced whey protein microgel particles, and TG enzyme cross-linked peanut protein isolate microgel particles all have the ability to stabilize HIPPE [9,33,34]. HIPPE stabilized by protein microgel particles, under the factors of pH, ionic strength, and particle concentration, can influence the properties of emulsion droplet size, stability, microstructure, rheological properties, etc. [31,35]. At the same time, HIPPE stabilized by protein microgel particles has a great potential for wide application in food. Jiao et al. [7], using peanut protein microgel particles, successfully made food-grade HIPPE (Figure 1) with an oil phase proportion up to 87%, which had similar spreadability and plasticity to that of margarine. In conclusion, compared with natural proteins, protein microgels can significantly increase the emulsion performance and stability of high internal phase emulsions. Constructing HIPPE based on protein microgel particles to serve as solid or semi-solid fat substitutes for some hydrogenated oil has a broad future.

In addition, protein hydrolysates have been considered as lotion stabilizers with great potential in recent years. The amino acids of protein hydrolysates expose hidden hydrophobic groups, improve the surface hydrophobicity, and reduce the molecular weight. At the same time, due to the high surface charge, they (protein hydrolysates) tend to be distributed at the oil–water interface in the lotion system, improving the stability of lotion. Lipid oxidation easily occurs at the oil–water interface. The protein hydrolysate adsorbed on the interface has the ability to scavenge free radicals and improve the oxidation stability of lotion. However, there are few reports on the application of protein hydrolysate in high internal phase Pickering lotion at present. In the future, protein hydrolysate can be used as a stabilizer of high internal phase Pickering lotion to further improve the oxidation stability of lotion.

### 2.2. Polysaccharide Granules

Polysaccharides are another type of commonly used solid particle stabilizers for HIPPE. Since most polysaccharides are hydrophilic, hydrophobic modification is usually needed to form stable emulsions. Commonly used polysaccharide particles are modified starch, cellulose derivatives, etc. (Table 2).

Starch, as hydrophilic macromolecules, can be prepared for hydrophobic modification by acid hydrolysis, enzyme hydrolysis, heat treatment, organic reagent modification, and complexation methods [36,37,38]. In addition, the esterification reaction of octenyl succinic anhydride (OSA) with starch is the most commonly used method for the preparation of modified starches. OSA–starch mainly consists of hydrophilic starch backbones with an amphiphilic nature by attaching to hydrophobic octenyl groups, which generates steric hindrance or electrostatic repulsion among the dispersed droplets to stabilize the emulsions [39], being a good high internal phase Pickering emulsion stabilizer [40]. Cellulose derivatives such as cellulose nanocrystals and bacterial cellulose can be irreversibly adsorbed at the oil–water interface to form fine droplets, thus developing into stable HIPPE [41,42]. HIPPE stabilized by cellulose derivatives can protect bioactive substances and, because of their high availability, non-toxicity, and biodegradability as effective food delivery systems, they have a great potential in the green food industry.

Apart from the two polysaccharides discussed above, other types of polysaccharide particles also have the ability to stabilize HIPPE. Chitosan is a natural polymer with non-toxic, biodegradable, and biocompatible properties and has good applications in the field of emulsions [43,44]. As well, pectin, gum arabic, and alginate all have good application characteristics in emulsion systems [45]. In the future, multiple methods will be used for the hydrophobic modification of polysaccharide particles to improve their emulsification ability, thus broadening the scope of application of food-grade HIPPE stabilized by more types of polysaccharides.

**Table 2:** Food-grade high internal phase emulsions stabilized by polysaccharides.

Particle Classification	Types of Polysaccharides	Particle Preparation Method	Emulsion Preparation Method	*Φ*	Ref.
Modified starch	Octenyl succinic anhydride (OSA)-modified starch	Add OSA into suspension and use NaOH to keep pH at 8–9; then, use HCl to adjust pH to 6.5 after the reaction. After centrifugation, clean with ethanol water and deionized water 3 times and prepare particles by drying and cooling.	Mix with oil and shear at 13,500 rpm for 3 min to prepare the emulsion.	74%	[40]
	Octenyl succinic anhydride (OSA)-modified starch	Add OSA into hydrolyzed starch solution, stir for 1 h under 35 °C, and use NaOH to keep pH at 8.5. After reaction, use HCl to adjust pH to 6.5. After centrifugation, clean with ethanol water and deionized water twice, dry in oven under 40 °C for 24 h, and then crush into granules by 100 mesh screen.	Mix with sunflower seed oil and shear at 23,000 rpm for 2 min to prepare the emulsion.	75%	[39]
	Hyperbranched polymers (HBPs)	Dissolve HBPs into N,N-Dimethylformamide (DMF); then, DMF is removed by dialysis after adding the polymers dropwise to distilled water to obtain particle suspension.	Mix with oil and shear at 4000 rpm for 2 min to prepare the emulsion.	80%	[46]
	Starch nanocrystals (SNC)	Mix starch with diluted H_2_SO_4,_ hydrolyze at 40 °C in a water bath with stirring, and then centrifuge and wash for 10 times. Finally, the suspension is further dialyzed in deionized water to remove the remaining H_2_SO_4_.	Mix with oil and shear at 13,500 rpm for 2 min to prepare the emulsion.	85%	[37]
Cellulose	Bacterial cellulose	Bacterial cellulose is homogenized by high pressure 40 times. Then, shear at 10,000 rpm for 15 min and further homogenize at 75 MPa. Finally, the suspension is concentrated by rotary evaporation.	Mix with oil and shear at 12,000 rpm for 2 min to prepare the emulsion.	75%	[47]
Chitosan	Carboxymethyl chitosan (CMCS)	Disperse CMCS in aqueous phosphoric acid solution overnight to fully hydrate.	Mix with oil and shear at 10,000 rpm for 30 s to prepare the emulsion.	82%	[48]
	Chitosan microgel (CS)	Prepare h-CS by hydrophobic modification of chitosan using deoxycholic acid and then prepare microgel particles through dispersing h-CS in ethanol aqueous solution by adding dropwise to sodium tripolyphosphate at 1000 rpm, stirring for 20 min, and then applying ultrasonic treatment.	Mix with oil and shear at 10,000 rpm for 2 min to prepare the emulsion.	85%	[49]
	Chitosan microgel (CS)	Dissolve CS in acetic acid and adjust pH to 5.5, filter through 0.45 μm filter membrane, and store at 4 °C. Finally, prepare microgels by mixing with genipin solution dissolved in ethanol in the water bath at 37 °C.	Mix with oil and shear at 10,000 rpm for 1 min to prepare the emulsion.	80%	[50]

### 2.3. Composite Particles

As stabilizers for food-grade HIPPE, proteins or polysaccharide particles usually need to be modified to have good stabilization due to their own poor emulsification ability. However, at present, the particles are usually modified using chemical methods, which do not meet the requirements of green food. The self-assembly of different particles can also achieve the same effect of emulsion stabilization.

Over the years, the particles have been mixed to produce different types of composite particles; there have been extensive studies on protein–polysaccharide composite particles, protein–protein composite particles, and polysaccharide–polysaccharide composite particles as the high internal phase Pickering emulsion stabilizers (Table 3). Self-assembled composite particles are significantly affected by the type of particles, concentration, dissolution temperature, pH, ionic strength, and the self-assembly methods [51,52]. Shen et al. [53] used the anti-solvent method with simple complexation to prepare protein–polysaccharide composite particles. Additionally, it was found that the particles prepared by the anti-solvent method had better protein conformation, surface activity, and gel strength, and the HIPPE stabilized with the anti-solvent composite particles had good droplet size, crystallinity, and thermal stability. Similarly, protein–protein composite particles prepared by the anti-solvent method had good wettability and aggregation properties and can be used to produce stable emulsions with high viscoelasticity [54].

The current research on composite particles mainly focuses on protein–polysaccharide composite particles, and the research on an emulsion system based on polysaccharide–polysaccharide composite particles’ stabilization is not sufficient for the time being. In-depth research can be conducted for composite particles in the future to enrich the application of different biomacromolecular particles in the health food industry. In addition, Janus particles prepared from biomacromolecules are also different from traditional particles in that they have anisotropic surfaces and exhibit interface surface activity and Pickering emulsion stabilizer capability [55]. Further studies in the future may prove that Janus particles have the ability to stabilize high internal phase emulsions.

**Table 3:** High internal phase emulsions stabilized by composites particles.

Particle Classification	Type of Composite Particles	Particle Preparation Method	Emulsion Preparation Method	*Φ*	Ref.
Protein–polysaccharides	Prolamin nanoparticles–gum arabic (GNPs/GA)	Dissolve protein particles in alcohol, stir continuously to nanoparticle suspension, rotate, and evaporate to remove alcohol; fully dissolve gum arabic in deionized water.	Mix GNPs’ solution with GA solution 1:1, add corn oil, and then shear at high speed for 2 min to prepare the emulsion.	85%	[51]
	Bacterial nanocellulose–soybean protein isolation (BCNs/SPI)	Mix SPI solution with BCNs’ solution, shear at 8000 rpm for 4 min, and then rotate to evaporate. Centrifuge to remove alcohol and excessive water to prepare particles.	Mix with sunflower seed oil and shear at 25,000 rpm for 3 min to prepare the emulsion.	75%	[53]
	Soybean protein isolation (SPI)–glucan	Stir SPI in deionized water for 2 h and then put it into the refrigerator for 24 h to fully hydrate. Freeze dry at −80 °C the mixed solution obtained by stirring the solution added with glucan.	Mix with corn oil and shear at 12,000 rpm for 1 min to prepare the emulsion.	74%	[56]
	Rice protein–carboxymethyl cellulose	Mix protein solution and carboxymethyl cellulose 1:1, adjust pH to 12.0, and then stir. Then, adjust pH to 7.0 and freeze dry through spin dialysis.	Mix with oil and shear at 13,000 rpm for 1 min to prepare the emulsion.	80%	[57]
	Prolamin–chitosan	Mix the protein dissolved in ethanol solution and the chitosan dissolved in acetum and homogenize them for 4 min at 6000 rpm. Prepare dispersions through rotary evaporation.	Mix with oil and shear at 20,000 rpm for 2 min to prepare the emulsion.	90%	[58]
	Lactoferrin–gum arabic	Mix lactoferrin with gum arabic solution in equal volume.	Mix with oil and shear at 1200 rpm for 2 min to prepare the emulsion.	75%	[52]
	Pea protein isolate–high methoxyl pectin (HMP)	Adjust the protein solution to pH 12.0, then heat it at 85 °C for 30 min, and immediately cool it to room temperature. Then, adjust the pH to 7.0 and compound it with HMP.	Mix with oil and shear at 15,000 rpm for 3 min to prepare the emulsion.	82%	[59]
Protein–proteins	Glycinin–flaxseed globulin	Stir the protein powder in water for 2 h respectively and add NaN_3_ to prevent bacterial growth. Heat protein solution 1:1 at 90 °C for 30 min.	Mix with oil and shear at 12,000 rpm for 1 min to prepare the emulsion.	85%	[60]
	Rice protein–walnut protein	Adjust the pH of the two protein solutions to 12.0 and store for 1 h. Mix the two solutions, stir for 1 h to adjust the pH to 7.0, and then freeze dry the solutions after spin dialysis.	Mix with oil and shear at 5000 rpm for 1 min to prepare the emulsion.	80%	[61]
Polysaccharide–polysaccharides	Chitosan–xanthan gum	Mix the two solutions, carry out ultrasonic treatment in an ice bath, and then further centrifuge the solution.	Mix with oil and shear at 10,000 rpm for 5 min homogeneously to prepare the emulsion.	85%	[62]

## 3. HIPPE Stability Mechanism

### 3.1. Particle Wettability

In the process of emulsion preparation, solid particles are wetted by both oil and water phases; the wettability of the particles is an important property to evaluate the emulsification ability of solid particles for HIPPE as well as the stability of the emulsion [53]. The wettability of the particles reflects their degree of hydrophilicity and hydrophobicity; only solid particles with dual wettability can be used as stabilizers for HIPPE. According to the different hydrophobic properties of particles, HIPPE can be divided into two types, oil-in-water (O/W) and water-in-oil (W/O).

The three-phase contact angle *θ* of solid particles at the oil–water interface is the most commonly used method to demonstrate wettability. The three-phase contact angle is the interfacial angle formed between the solid particles, oil phase, and water phase, which is usually expressed by Young’s formula:

$$\cos\theta =\frac{y_{so}-y_{sw}}{y_{ow}}$$

where $y_{so}$ is the interfacial tension between the solid particles and the oil phase, $y_{sw}$ is the interfacial tension between the solid particles and the water phase, and $y_{ow}$ is the interfacial tension between the oil and water phases.

**Figure 2:**
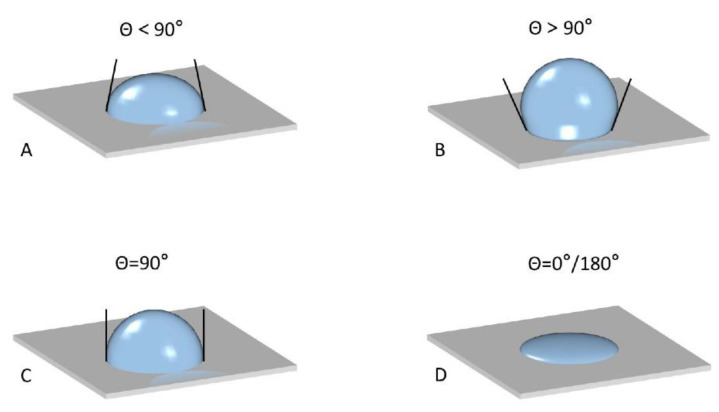
Schematic diagram of three-phase antenna. (**A**) *θ* < 90°; (**B**) *θ* > 90°; (**C**)*θ* *=* 90°; (**D**) *θ* *=* 0°/180°

When *θ* < 90°, the solid particles are immersed in the aqueous phase, being hydrophilic and able to form oil-in-water (O/W) emulsions [63]. When *θ* > 90°, the solid particles are hydrophobic, which will form water-in-oil (W/O) emulsions [64]. When *θ* = 90°, the solid particles are amphiphilic and can effectively adsorb on the droplet surface, forming steric hindrance to inhibit the aggregation of oil droplets in the emulsion, thus forming the most stable Pickering emulsions [65]. When *θ* is 0° or 180°, the solid particles become extremely hydrophilic or hydrophobic, which cannot form stable emulsions (Figure 2). Meanwhile, the *θ* varies at different pH. The solid particles are hydrophobic and hydrophilic under acidic and alkaline conditions, respectively. Furthermore, the influence of particle size on the three-phase contact angle *θ* also has a significant effect: a smaller particle size provides better wetness [49,66].

According to the above mechanism, Chen et al. [67] found that, due to the poor wettability of ovalbumin and protein aggregation at the isoelectric point *θ* lower (about 61.7°), by adding tannic acid, due to hydrogen bonding, the *θ* increased to around 90° and stable high internal phase Pickering emulsion was formed. Li et al. [68] similarly found that, with chitosan adsorption on a barley protein surface via hydrogen bonding and hydrophobic interaction, *θ* changed from 116.5° to 89.3°, which improved particle hydrophilicity. To sum up, the solid particles can be compounded or the surface can be modified to improve the contact angle of the particles in three phases up to about 90° to form stable high internal phase Pickering emulsion.

### 3.2. Interfacial Membrane Adsorption

**Figure 3:**
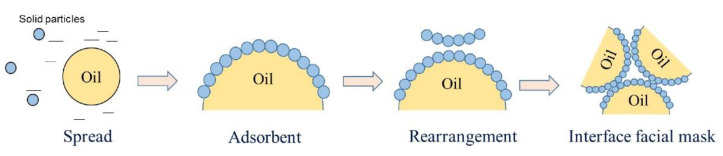
Interface film formation mechanism based on diffusion–adsorption–rearrangement mechanism.

The existent form and aggregation state of food macromolecule particles at the O/W interface directly affect the stability and processing performance of HIPPE. The recognized stabilization mechanism of Pickering emulsions is that the solid particles diffuse due to hydrophilic and hydrophobic properties that then adsorb at the O/W interface. Then, after structural rearrangement, solid particle monolayers or multilayers are formed (Figure 3), which gradually reduce the system free energy and stabilize the emulsion [69,70].

The interfacial properties of the particles have an important influence on the emulsification performance. After the oil phase contacts with the water phase, the particles quickly adsorb to the oil–water interface at first; then, the interface gradually fills up over time, resulting in the interfacial tension showing a gradually decreasing trend [71]. The interfacial properties are affected by particle wettability, pH, particle size, and interfacial film thickness [72]. Particles with higher hydrophobicity have better interfacial activity [73], while the interfacial tension decreases gradually with a decreasing pH **[50]**. Compounding improves the surface wettability and steric hindrance of the particles, forming a denser interfacial film and preventing the aggregation of oil droplets [74]. In the future, by studying the solid particle assembly and the interfacial membrane with adsorption behavior, the mechanism of high internal phase Pickering emulsion stabilization will be clarified.

With the deepening of research, it has been found that HIPPE have problems such as low water phase ratio, large droplet deformation (hexagonal shape), thin phase interface layer, etc. There is no space for structural rearrangement of particles after diffusion and adsorption. The traditional “diffusion–adsorption–rearrangement” theory can no longer perfectly explain the formation mechanism of oil–water interfacial film; so, it is necessary to enrich and improve the new theory of solid particle adsorption during the formation of HIPPE.

### 3.3. Dual Network Structure

In recent years, the dual network structure has been brought up as an advanced mechanism to stabilize HIPPE; the gel network has a more stable and higher viscosity structure that can stabilize HIPPE [36]. In this case, the solid particles are not necessarily irreversibly adsorbed at the oil–water interface and may act as structuring agents to form very stable emulsions [75]. Tao et al. [76] co-stabilized HIPPE with zein protein nanoparticles (ZNPs) and starch nanoparticles (SNCs), and a unique self-assembled bilayer structure was formed among the particles. The particle bilayer structure consisting of the inner adsorbed ZNPs and the outer adsorbed SNCs could provide better stability, and the prepared HIPPE could replace commercially available mayonnaise and salad dressing in terms of appearance and performance. Apart from the dual network structure, novel stabilization mechanisms of HIPPE such as interparticle capillary forces and interfacial rheological response have been reported [75]. However, these mechanisms are not sufficiently studied at present, and more investigations on the stabilization mechanism of HIPPE can be carried out in the future.

## 4. Application of HIPPE in 3D Printing

A healthy and fashionable diet has gradually become the new goal of modern people. With the advantages of operability and convenience, 3D printing of food is very likely to become an indispensable part of modern human life.

### 4.1. Food 3D Printing Technology

The 3D printing technology, as one of the products of modern technological development, is widely used in industrial production, biomedicine, and food [77,78]. Food 3D printing technology is a new food processing technology that can personalize the ratio of ingredients and the appearance for different people [79]. Additionally, by making the easy-to-take food textures, it can provide specific nutrients for elderly people, as well as customized food for children, pregnant women, and athletes with appropriate nutritional needs [80]. There are three main printing technologies for food 3D printing including the extrusion-type printing, powder condensation-type printing, and inkjet printing, among which the most commonly seen is the extrusion-type printing. The extrusion process is digitally controlled, and a 3D food product will be finally obtained by printing layer by layer according to the set path [81].

**Figure 4:**
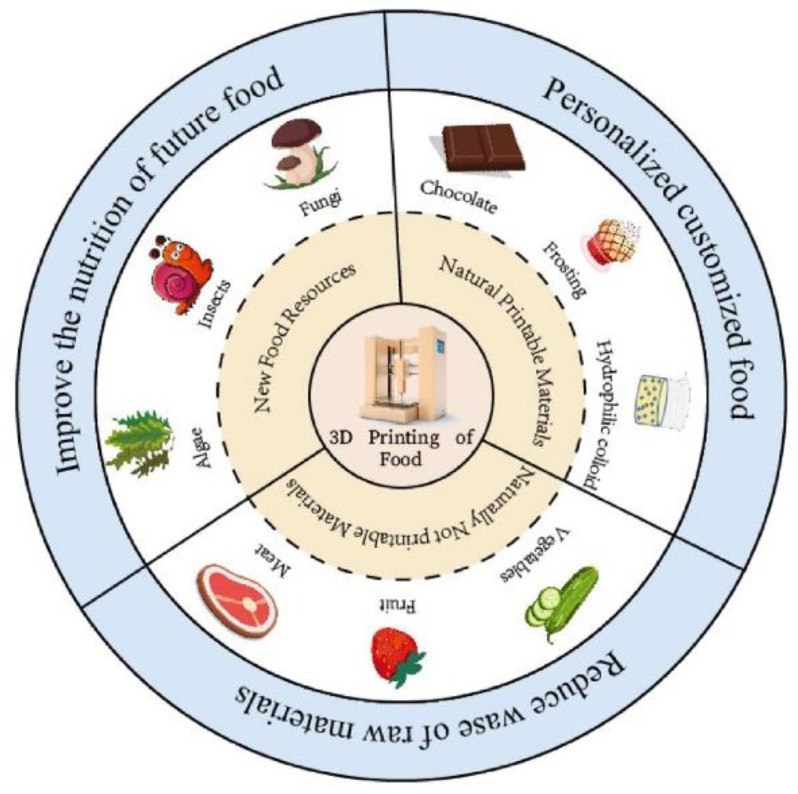
Advantages and raw materials of food 3D printing.

The raw materials in food 3D printing are divided into three types: natural printable raw materials, natural non-printable raw materials, and advanced food resources (Figure 4). The natural, printable materials have excellent printing properties and can be smoothly extruded through the print spout; the commonly seen materials include chocolate, frosting, and hydrocolloids. The natural, non-printable raw materials need to be processed to enhance extrusion and textural properties to meet printing needs; these materials include meat, fruits, and vegetables. The advanced food resources can be used as substitutes for traditional foods to meet the requirements of a healthy diet; these include algae and insects that are rich in protein and fiber [82].

At present, 4D printing could be developed by adding a time dimension to 3D printing, and the color and shape of food products will change with time [83], while 5D and 6D printing, as a newly emerging technology, can print more complex foods with fewer raw materials. Future directions for these new technologies include realizing high-quality foods that cannot currently be produced, bringing major innovation to the food field, and greatly increasing the interesting and educational significance of 3D printed foods.

### 4.2. Key Technologies for Food 3D Printing

Food printing is divided into four stages of formula, model design, 3D printing, and post-processing. The characteristics of printing material such as rheological properties, extrusion method, and printing parameters such as printing temperature, printing distance, etc. can all affect the effect of 3D printing.

The ink should not only be easily extruded through the nozzle but also have sufficient mechanical strength to reduce deformation [84]. The rheology of food ink is a major parameter affecting the extrusion-type 3D printing technique and is critical to the printability [85,86]; the non-Newtonian fluid with shear thinning behavior has excellent printability [82]. The printing temperature affects the rheological properties of food products, which, in turn, affect the printability of the ink for 3D printing as well as the hardness, elasticity, and chewiness of products [87,88]. The extrusion method of 3D printing can significantly affect the quality of the products. The common extrusion methods are syringe type, pneumatic type, and screw type. The appropriate extrusion method should be selected by comprehensively considering the flowing properties and thermal characteristics as well as other factors of food materials, so as to better improve the quality of the printed products. Printing parameters such as nozzle size, nozzle movement speed, and nozzle height all have a significant impact on the printed products. Improper printing parameters may cause deviations in the printed product, failing to print corner sections [89], and cause errors due to delayed deposition [90]. In summary, printing parameters are of great value in the quality assessment of 3D printed products, and printing food products with personalized nutrition by choosing the right parameters is a popular trend for the future.

### 4.3. The 3D Printing of HIPPE

In recent years, HIPPE has been used in the 3D printing of porous materials [91]. Food-grade high internal phase emulsions tend to have high viscosity and stability and can be used as good food inks for printing foods in special shapes through nozzle extrusion such as letters, apples, and turtles. Rheological properties and printing parameters are the most important factors affecting the 3D printing of HIPPE.

As pseudoplastic non-Newtonian fluids with shear thinning properties, HIPPE can be smoothly extruded from the nozzles of 3D printers while being sufficiently viscous to maintain the resolution of the printed shapes [92,93]. In the process of 3D printing, the concentration and type of solid particles affect the stability and rheology of the emulsions as well as the resolution of printed products [57]. Zhang et al. used sea bass protein microgel particles to stabilize HIPPE loaded with astaxanthin and found that a high concentration of protein could result in a stronger viscoelasticity and excellent thixotropy of the emulsion [94]. Ma et al. [95] used 0.5 wt.% concentration of cellulose nanocrystals to stabilize HIPPE to obtain good rheological properties, while the products printed at higher particle concentrations had poor resolution. In contrast, composites of different solid particles can adjust the wettability, reduce the interfacial tension, and provide good steric hindrance and electrostatic repulsion, providing HIPPE good gel properties and surface viscosity. Additionally, just because of this, the 3D printing products have excellent resolution, hardness, viscosity, and chewiness [57]. Moreover, the oil phase also has a significant effect on the emulsions and products. Feng et al. [96] used cinnamaldehyde and tea seed oil as the mixed oil phase for HIPPE and found that the structural strength and viscoelasticity of the emulsions decreased with the increase in cinnamaldehyde content, while the resolution and structural stability of the printed products also decreased, with the products showing a bad state of surface roughness and structural collapse. The mechanical strength can be expressed by the energy storage modulus (G’). A higher G’ can provide better printing accuracy and product resolution, improve the appearance of the product, contribute to structural stability, and keep the product in shape, while a lower G’ will cause the printed products to collapse and make the structure incomplete due to an insufficient mechanical strength to support the structure. Therefore, only by characterizing the rheological properties of the emulsion before printing and choosing a suitable G’ can we make the smooth extrusion of emulsions from the fine nozzle with sufficient strength to make product shapes. In addition, ionic strength, pH, and other factors can also have an effect on the rheological properties of HIPPE, which further affects the 3D printing effect.

Printing parameters such as nozzle diameter, nozzle height, and nozzle movement speed also have a significant impact on HIPPE-based 3D printing. Smaller nozzle sizes have a good effect on the dimensional performance of HIPPE-based 3D printed products. Larger sizes will lead to deviations in the printed products, but the undersized nozzles will cause inconsistencies in the length and width of HIPPE during extrusion, resulting in undesirable effects. The height of the nozzle should be in the lowest possible range. A lower height will avoid errors caused by delayed deposition. At the same time, the nozzle should not move too fast, since faster speeds may cause the nozzle to drag the extruded materials, resulting in product surface breakage and the corner sections possibly not being printed successfully. In addition, the printing temperature is also an important factor affecting the printing results. Currently, the printing is often conducted at room temperature (25 °C). In addition, the printing temperature is also an important factor affecting the printing results. Setting the appropriate printing parameters is beneficial for printing food-grade HIPPE into special shapes (Table 4).

If HIPPE is applied to food 3D printing, it is not enough to only study whether HIPPE can be successfully extruded through the nozzle and factors of appearance and nutrition; the shelf life of the printed product also needs to be considered. Current research shows that the increase in particle concentration can improve the oxidation stability of lotion, which is conducive for the application of a Pickering lotion system with high internal phase in food. Therefore, in the future, microbial growth could be inhibited by adding salts to HIPPE, and, through encapsulating pigments such as lutein and carotene, the products will have rich colors. We could enhance the nutrition of products by utilizing the property of HIPPE loaded with nutrition. The oxidation stability of the lotion system was investigated, thus further exploring the potential of HIPPE as 3D printing food ink.

**Figure 5:**
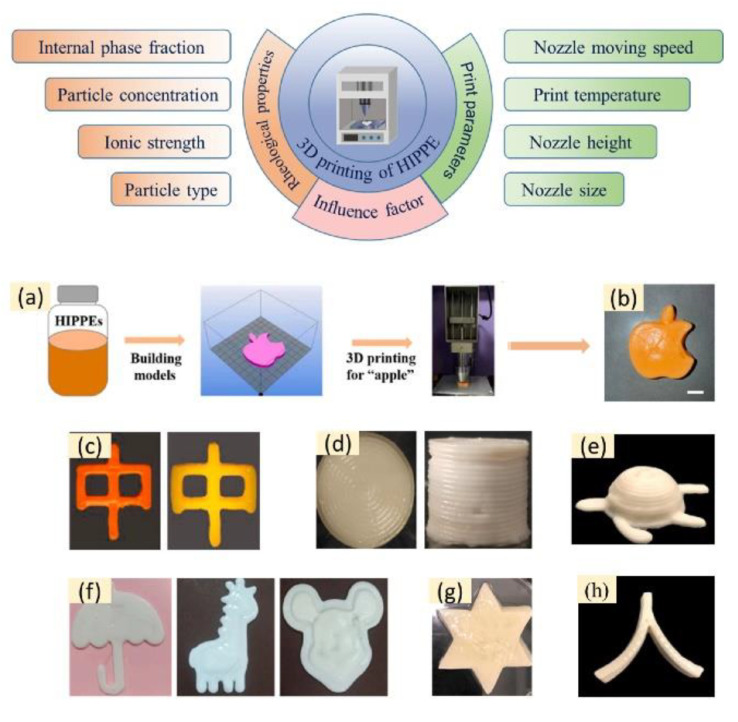
Factors and products influencing HIPPE-based 3D printing. (**a**,**b**) The 3D printing process and samples of high internal phase emulsion based on sea bass protein microgel, reprinted with permission from Ref. [94]. Copyright 2022 Elsevier; (**c**) gelatin-based HIPPE, reprinted with permission from Ref. [97]. Copyright 2021 Elsevier; (**d**) HIPPE based on casein/pectin composite particles, reprinted with permission from Ref. [92]. Copyright 2022 Elsevier; (**e**,**h**) 3D printing of a turtle shape based on HPPE, reprinted with permission from Ref. [66]. Copyright 2022 Elsevier; (**f**) gelatin-based HIPPE, reprinted with permission from Ref. [93]. Copyright 2021 Elsevier; (**g**) star model based on HIPPE, reprinted from Ref. [98].

Based on the above discussions, we applied food-grade HIPPE with good viscoelasticity and thixotropy to 3D printing. Firstly, different 3D solid models were designed. Then, layer by layer, they were printed through the set printing program and the settings and stacked, thus printing food of various shapes (Figure 5a,b). Studies have shown that due to its excellent injectability, high G’ and yield stress, and low frequency dependence, HIPPE has good printable properties. HIPPE based on casein and pectin composite particles has higher viscosity, mechanical strength, and gel strength due to the addition of pectin, allowing it to print well-shaped cylinders that can avoid the collapse of the product at higher concentrations of pectin content (Figure 5d). HIPPE based on gelatin has high elasticity, thixotropy, and recovery rate and its good extrusion capability and printing performance allow it to print a variety of shapes according to the set model (Figure 5f).

The 3D printing is widely applied in the food sector and is becoming a research hotspot in the food industry, although there is a lack of HIPPE-based 3D printing research, especially on the use of plant proteins as solid particle stabilizers. Research on 3D printing of food-grade HIPPE with the widely available and low-cost vegetable proteins as stabilizers is an important direction for future research.

## 5. Conclusions

Solid biological particles with good amphiphilicity, adsorption on the oil-water interface film can be irreversibly performed to stabilize the food-grade HIPPE. Currently, the most widely used macromolecular biological particles are proteins, polysaccharides, and their composite particles. The wettability can be improved by adjusting the contact angle of particles to about 90°, which is conducive to the formation of more stable emulsions. The high internal phase Pickering emulsion has a high-energy storage modulus as a non-Newtonian shear thinning fluid, which allows it to smoothly pass through the nozzle foundation and maintain a good shape. It has broad application prospects in the field of 3D printing of food, but some problems still need to be solved. Firstly, at present, particle research is mainly focused on plant protein and there are few reports on meat protein-stabilized emulsion. Most composite particle selection is performed on protein–polysaccharides. It is not enough to report whether other types of composite particles can well stabilize HIPPE. At the same time, the modification of particles is usually by chemical modification, which does not meet the requirements of green food. Secondly, traditional diffusion adsorption rearrangement theory cannot perfectly explain the formation and stability of the oil–water interface of HIPPE, so there is an urgent need to enrich and perfect the theory of it. Finally, research on 3D food printing based on HIPPE is not enough. At present, it mainly focuses on the rheological properties of emulsion and the printing effect of HIPPE; there are few reports on the application of HIPPE to real food systems.

Therefore, future research on food-grade high internal phase Pickering emulsions could focus on the following aspects: (1) through the compounding of more types of biomacromolecular particles, such as protein–protein and polysaccharide–polysaccharide composite particles, to explore whether they have the potential to stabilize high internal phase emulsions; (2) to investigate the multi-scale structure of the oil–water interface, to construct the theory of the dynamic formation of the emulsion structure from a microscopic view to a macroscopic view and from the interface to the bulk phase, and to explore the advanced stabilization mechanism of HIPPE; (3) to inspect the rheological properties and stability of high internal phase emulsions and, by taking into account factors such as nutrition, flavor, color, and shape, to develop the 3D printing of easy-to-swallow and specially shaped foods, thus expanding the application prospects of HIPPE in the food industry.

## Figures and Tables

**Table 4:** HIPPE –based 3D printing parameters and shapes.

Solid Particles	*Φ*	Printing Parameters	Shapes	Ref.
Gelatin/chitosan oligosaccharide	75%	Nozzle diameter 1.2 mm, nozzle height 1 mm, movement speed 20 mm/s, temperature 25 °C.	Heart shape, umbrella-shape, deer, clover, etc.	[93]
Sea bass protein microgel particles	88%	Nozzle diameter 1 mm, movement speed 1 mm/s, temperature 25 °C.	Apple	[94]
Rice protein/ carboxymethyl cellulose	85%	Nozzle diameter 0.8 mm, nozzle height 0.8 mm, movement speed 22 mm/s, temperature 25 °C.	Cylinder	[57]
Gelatin	75%	Nozzle diameter 2 mm, nozzle height 1 mm, movement speed 20 mm/s, temperature 25 °C.	Chinese characters	[97]
Protein/polysaccharide	80%	Nozzle diameter 1.2 mm, movement speed 900 mm/s, temperature 25 °C.	Turtle	[66]
Cellulose nanocrystal	80%	Nozzle diameter 0.51 mm, nozzle height 0.51 mm, temperature 25 °C.	Letters	[95]

## Data Availability

Not applicable.
